# *Rpv* Mediated Defense Responses in Grapevine Offspring Resistant to *Plasmopara viticola*

**DOI:** 10.3390/plants9060781

**Published:** 2020-06-22

**Authors:** Tyrone Possamai, Daniele Migliaro, Massimo Gardiman, Riccardo Velasco, Barbara De Nardi

**Affiliations:** 1 Department of Agricultural, Food, Environmental and Animal Sciences, University of Udine, via delle Scienze 206, 33100 Udine, Italy; possamai.tyrone@spes.uniud.it; 2 CREA - Research Centre for Viticulture and Enology, viale XXVIII Aprile 26, 31015 Conegliano (TV), Italy; massimo.gardiman@crea.gov.it (M.G.); riccardo.velasco@crea.gov.it (R.V.); barbara.denardi@crea.gov.it (B.D.N.)

**Keywords:** grape breeding, sporulation, necrosis, leaf discs, downy mildew, pyramiding, *Rpv*

## Abstract

Downy mildew, caused by the biotrophic oomycete *Plasmopara viticola*, is one of the most serious grapevine diseases. The development of new varieties, showing partial resistance to downy mildew, through traditional breeding provides a sustainable and effective solution for disease management. Marker-assisted-selection (MAS) provide fast and cost-effective genotyping methods, but phenotyping remains necessary to characterize the host–pathogen interaction and assess the effective resistance level of new varieties as well as to validate MAS selection. In this study, the *Rpv* mediated defense responses were investigated in 31 genotypes, encompassing susceptible and resistant varieties and 26 seedlings, following inoculation of leaf discs with *P. viticola*. The offspring differed in *Rpv* loci inherited (none, one or two): *Rpv3-3* and *Rpv10* from Solaris and *Rpv3-1* and *Rpv12* from Kozma 20-3. To improve the assessment of different resistance responses, pathogen reaction (sporulation) and host reaction (necrosis) were scored separately as independent features. They were differently expressed depending on *Rpv* locus: offspring carrying *Rpv3-1* and *Rpv12* loci showed the strongest resistance response (scarce sporulation and necrosis), those carrying *Rpv3-3* locus showed the highest levels of necrosis while *Rpv10* carrying genotypes showed intermediate levels of both sporulation and necrosis.

## 1. Introduction

Downy mildew (DM) caused by the obligate oomycete *Plasmopara viticola* (Berk. & Curt.) Berl. & de Toni is one of the most destructive grapevine diseases occurring worldwide. *Vitis vinifera* varieties are highly susceptible to fungus infections, leading to serious quantitative and qualitative yield reductions in vineyards.

On the contrary, several *Vitis* species showing variable levels of resistance to DM and mechanisms of disease control have been identified in North America (e.g., *M. rotundifolia*, *V. rupestris*, *V. labrusca*, *V. riparia*, *V. cinerea*) and Asia (e.g., *V. amurensis*, *V. piasezkii*, *V. coignetiae*) [1,2].

To date, 27 quantitative trait loci (QTL) associated with *P. viticola* resistance (*Rpv*) have been identified [3]. The *Rpv3* locus originated from North American species was mapped on chromosome 18. It is characterized by multiple resistance alleles or paralogues, which have been conserved by the human intervention [4]. The resistance haplotype *Rpv3-1* is the most frequent in selected resistant varieties and it has been observed in Villard blanc, Bianca [5], Kozma 20-3 [6] and Regent [7,8]. Other haplotypes are less widespread in breeding selections, such as *Rpv3-3*, which has been maintained for example in Seyval, Merzling and Solaris [4,9]. *Rpv10* and *Rpv12* resistance loci originated from the Asian species *V. amurensis: Rpv10* locus was mapped on chromosome 9 from Solaris [10], while *Rpv12* on chromosome 14 from Kozma 20-3 [6].

The resistance traits to downy mildew have been introgressed into *V. vinifera* background through interspecific crosses since the second half of the 1800s. Several fungus-resistant selections were developed carrying different *Rpv* loci by backcrossing the best selected parental lines several times with *V. vinifera* elite varieties, thus considerably improving the grape properties of the new materials. With the utilization of genotyping strategies, such as marker-assisted selection (MAS), the breeding process has been much improved. Nowadays, MAS is effectively used for early discrimination of genotypes carrying different resistance sources as well as individuals with pyramided favorable alleles for the same resistance trait [11].

The accurate evaluation of disease symptoms is crucial to understand the genetic factors underlying resistance to plant pathogens, as well as to identify associated markers and investigate interactions. Grape leaf degree of resistance to *P. viticola*, in the field and on leaf discs, is traditionally performed according to the Organisation Internationale de la Vigne et du Vin (OIV) descriptors 452 and 452–1 [12]. The OIV scales classify the degree of resistance to *P. viticola* considering the fungus sporulation and plant necrosis together. Recently, other classification protocols have been proposed improving the possibility for genotype discrimination when the plant necrotic response and pathogen sporulation are considered separately [13].

In this study, we report the molecular (MAS) and phenotypic (bioassay) characterizations of a representative set of susceptible and resistant genotypes belonging to the CREA - Research Centre for Viticulture and Enology breeding program. The aim is to evaluate the defense response to *P. viticola* in genotypes carrying different *Rpv* loci (*Rpv3-1*, *Rpv3-3*, *Rpv10* and *Rpv12*).

## 2. Results

### 2.1. Segregating Populations and MAS

The *V. vinifera* Raboso Piave variety was crossed with the grape hybrids Kozma 20-3 and Solaris carrying different resistance genes to *P. viticola* (Table S1). Two populations of 224 Raboso Piave x Kozma 20-3 (RPxK) and 538 Raboso Piave x Solaris (RPxS) individuals were obtained, respectively.

All the progenies were screened for the specific segregating *Rpv* loci using SSR markers (Table S1). Genotypes carrying at least one resistance haplotype (*Rpv+*) as well as a few individuals with no genetic resistance (*Rpv-*) were retained. In particular, 110 RPxK genotypes carrying the resistance alleles of *Rpv3-1*, *Rpv12* or both and 255 RPxS seedlings having the resistance alleles of *Rpv3-3*, *Rpv10* or both, were maintained (Table S2). Based on the presence of the respective resistance alleles, 7 *Rpv* classes were considered. A total of 26 progenies differing in the carried resistance sources, the parental plants and the susceptible varieties Chardonnay and Glera were assessed for their resistance to downy mildew by leaf disc bioassay.

### 2.2. Phenotyping

#### 2.2.1. Discs Scores

**Figure 1:**
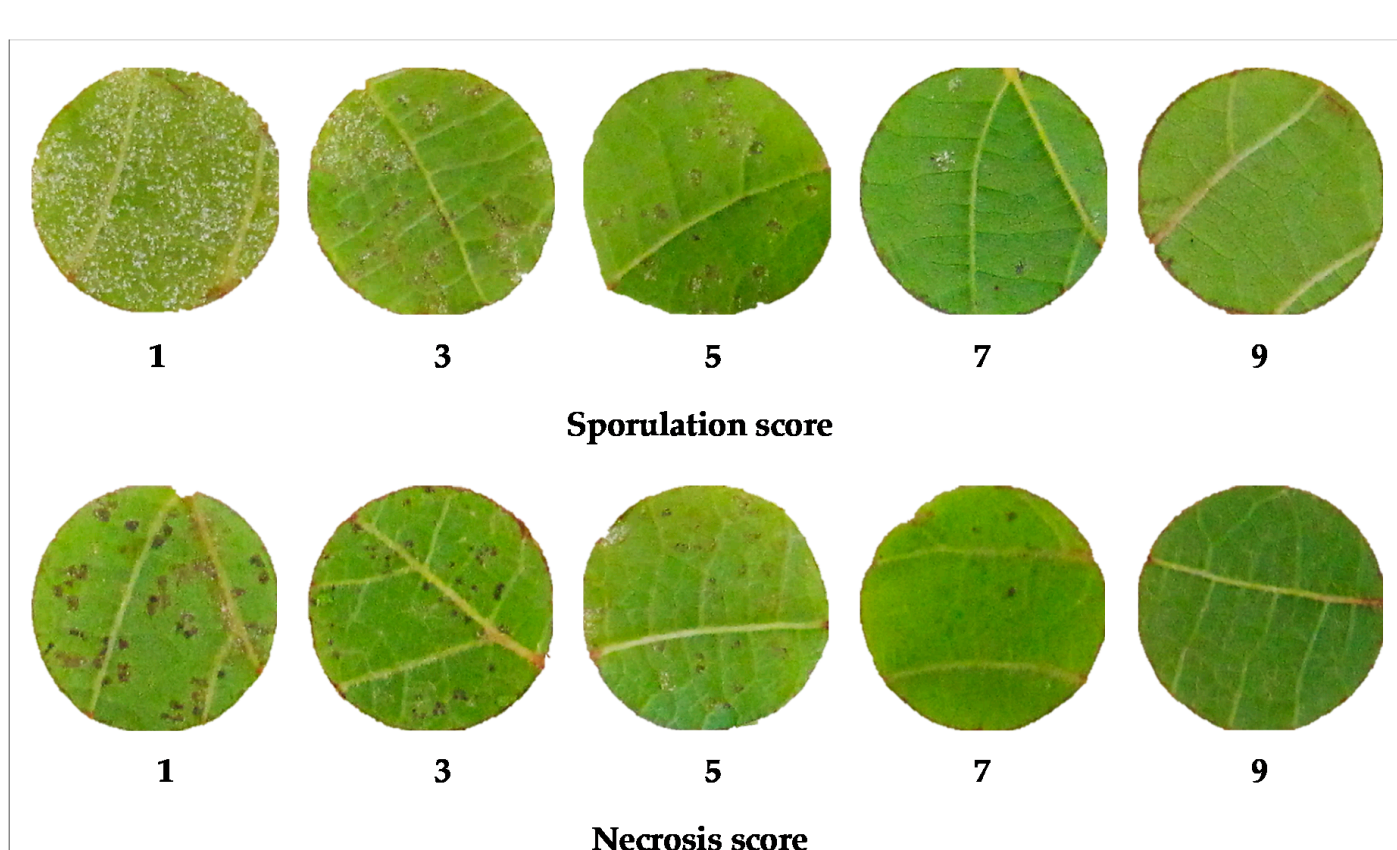
Dual-scale classification of symptoms. Sporulation and necrosis were evaluated separately by visual inspection of each leaf disc at 9 days post-inoculation with *P. viticola*.

Downy mildew infection was evaluated on leaf discs in two independent experiments collecting a total of 372 records. Disease symptoms were scored at 9 days-post-inoculation (dpi): sporulation and necrosis were assessed separately with two scales (Figure 1).

**Figure 2:**
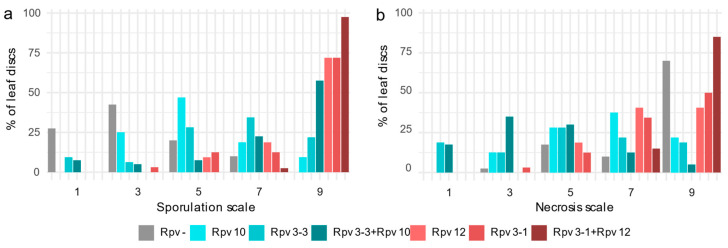
Leaf disc classification according to the dual-scale system. Sporulation and necrosis rating are reported in panels “a” and “b”, respectively. Shades of blue identify the progenies of Solaris (RPxS). Shades of red the progenies of Kozma 20-3 (RPxK). Gray represents the *Rpv-* genotypes.

Concerning sporulation, discs belonging to the two populations were separated into symptom severity classes and clear differences were observed with regard to *Rpv* profile (Figure 2, panel a). Progenies derived from Kozma 20-3 showed poor or absent sporulation (scores 7 to 9) when they had one resistance source (*Rpv3-1* or *Rpv12*) and, in particular, when they had two combined loci (*Rpv3-1*+*Rpv12*). Otherwise, F1s of Solaris, showed a higher severity of symptoms on average, including genotypes with a score of 3, and in all cases more sporulation than the resistant parent. When *Rpv3-3* and *Rpv10* sources of resistance were combined, *P. viticola* growth was less relevant than the sporulation revealed on progenies carrying a single *Rpv3-3* or *Rpv10* locus. All the discs of the progenies of *Rpv-* class showed high incidence of sporangia (score 3 and 1).

Regarding necrosis, as observed for sporulation, different type and extent of symptoms were observed among genotypes belonging to different populations and/or *Rpv* classes (Figure 2, panel b). In particular, most of the discs of “RPxK” population with *Rpv3-1* and *Rpv12* alleles, were distributed in the classes 7 and 9 (few or no necrotic spots), while discs of seedlings carrying *Rpv3-3* or *Rpv10* resistance alleles exhibited even severe necrotic responses (scores 5 to 1). Indeed, the genotypes combining *Rpv3-3* and *Rpv10* showed the highest incidence of necrotic symptoms.

Different symptomatic profiles were obtained for the analyzed genotypes combining sporulation and necrosis discs scores (Figure S1). Discs from the same genotype normally exhibited differences in scoring not exceeding two classes, except for a few cases that showed a more variable response against *P. viticola*. The susceptible varieties Raboso Piave, Chardonnay and Glera showed a dissimilar phenotype compared to Kozma 20-3 and Solaris; *P. viticola* was able to sporulate on each leaf disc of the susceptible group, while sporangia were not observable on the two resistant parents. Evidence of necrosis was present only on one-third of discs of the susceptible varieties, with a varying severity of the symptoms and mainly on very sporulated discs (scores 1 to 3). The resistant varieties differed in necrotic reaction: Solaris developed necrosis with intermediate intensity (scores 5 to 7); Kozma 20-3 had no visible response (score 9) on most of the discs or only a few necrotic spots (score 7).

Concerning the progenies, plants belonging to “*Rpv3-1*+*Rpv12*” class showed phenotype symptoms and scores comparable to Kozma 20-3. Indeed, progenies having only *Rpv3-1* or *Rpv12* showed scarce sporulation and necrosis (scores 7 and 9) on many discs. On the contrary, “*Rpv3-3*+*Rpv10*” carrying F1s, except for one genotype (RPxS_027) that exhibited a susceptible-like phenotype, showed limited sporulation like Solaris, but higher necrotic response compared to their parent. Plants with either *Rpv3-3* or *Rpv10* resistance alleles always displayed a higher sporulation level than the parent. In addition, *Rpv3-3* class genotypes had the more varied intensity of symptoms.

#### 2.2.2. Sporulation and Necrosis Correlation

The sporulation and necrosis scores of a disc did not differ in more than one class for 65% of samples, in particular in the case of scores in class 7 and 9 (e.g., genotypes RPxK_026) while in 35% of samples either sporulation or necrosis was predominant (e.g., genotypes RPxS_036). Intense sporulation and necrosis were rarely observed together; the score 1 for both was not recorded at all.

The observations suggested an absence of a general correlation between sporulation and necrosis, which was confirmed by the Kendall correlation coefficient TAU of 0.15.

Looking at the correlation among sporulation and necrosis scores within *Rpv* classes, the highest coefficients, with a statistically significant value (*p* < 0.05), were found in discs with *Rpv3-1* (TAU 0.65), *Rpv3-1*+*Rpv12* (TAU 0.47) and *Rpv3-3*+*Rpv10* (TAU -0.46). Instead, discs of genotypes carrying *Rpv3-3* did not show a significant correlation between sporulation and necrosis.

#### 2.2.3. Symptomatic Profile of Different *Rpv* Classes

Differences in symptomatology among the *Rpv* classes were investigated by fitting two linear mixed effect models (LMM), one for pathogen sporulation and one for plant necrotic response. The average scores from the four discs of the same genotype per experiment were used in the data analysis (Table S3). Interaction between the fixed factors “*Rpv* class” and “experiment” was not significant and was excluded from the LMMs.

**Figure 3:**
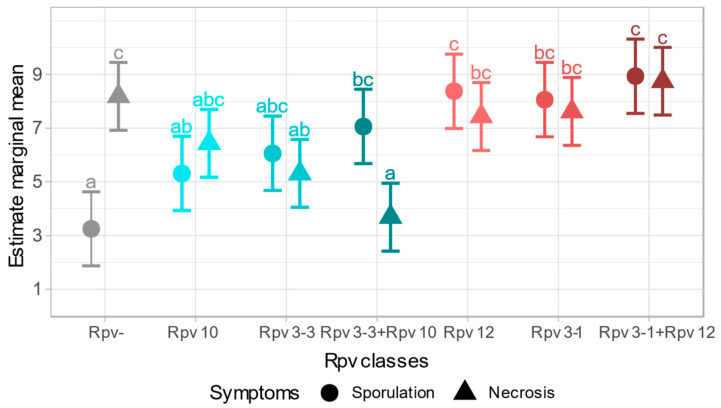
Estimated marginal means calculated by linear mixed models for both sporulation and necrosis reactions. Shades of blue identify the progenies of Solaris (RPxS). Shades of red the progenies of Kozma 20-3 (RPxK). Letters indicate statistically different symptoms among *Rpv* classes, obtained by pairwise comparisons (Tukey method). Bars indicate standard errors.

In the LMM model fitted for sporulation, the *Rpv-* class showed the lowest score; instead, *Rpv+* classes gave higher values. Sporulation in experiment A was lower than in experiment B, with score of 0.88. Variability due to the random effect “genotype” explained half of the variability not directly investigated with the LMM and its value (1.10) was similar to the residual error variability. The “*Rpv* class” and “experiment” factors both resulted both significant (*p* < 0.001 and *p* < 0.01, respectively). The pairwise comparison (Figure 3) showed that the selected classes *Rpv3-1*, *Rpv12*, *Rpv3-1*+*Rpv12* and *Rpv3-3*+*Rpv10* had a different sporulation reaction compared to susceptible plants (a = 95%). In contrast, plants with only the *Rpv3-3* or *Rpv10* loci showed a mean level of sporulation not statistically different from *Rpv-* plants. A significantly different reaction was also observed in the resistance *Rpv10* class compared to *Rpv12* and *Rpv3-1*+*Rpv12*.

In the LMM fitted for the necrotic reaction, the *Rpv-* class did not show the lowest score and its estimate mean had an intermediate value compared to the other *Rpv* classes. The necrosis estimate mean in experiment B was higher than in experiment A, with a score of 0.34. Standard deviation attributed to the genotype in this model was 0.56 and was much lower than the residual error variability calculated as 1.53 points. The model identified “*Rpv* class” as the only significant factor (*p* < 0.001), the difference between experiments resulted not significant (*p* = 0.41). From pairwise comparison, plants with the *Rpv3-3* locus, with or without *Rpv10*, exhibited statistically different necrosis expression from plants with no resistance loci (*p* > 0.05). The class *Rpv3-3*+*Rpv10* showed a higher necrotic response and was also significantly different from *Rpv3-1*, *Rpv12* and *Rpv3-1*+*Rpv12* classes.

In sporulation LMM, *Rpv3-1*+*Rpv12* and *Rpv3-3*+*Rpv10* classes showed a lower sporulation degree compared to the respective single locus. Concerning necrosis, plants with *Rpv3-1*+*Rpv12* were the least symptomatic, while the genotypes with *Rpv3-3*+*Rpv10* were the most symptomatic. However, pyramiding of the loci, *Rpv3-1* with *Rpv12* and *Rpv3-3* with *Rpv10*, did not provide significantly different phenotypes compared to the single relative *Rpv* classes. Nevertheless, a synergic loci effect seemed likely to be present and, in some cases, determined a significant difference between *Rpv* classes. Details of the two fitted LMM are reported in Table S4.

## 3. Discussion

In this study, two populations that segregated for *Rpv3-1* and *Rpv12* (Raboso Piave x Kozma 20-3) and for *Rpv3-3* and *Rpv10* (Raboso Piave x Solaris) were genotyped by SSR markers and a subset of genotypes carrying different *Rpv* loci was artificially infected with *P. viticola*. Parents as well as the *V. vinifera* susceptible varieties Chardonnay and Glera were also analyzed.

In general, leaf discs from the same genotype exhibited analogous symptoms. Indeed, F1s carrying the same resistance alleles showed quite similar phenotypes. Phenotypic evaluation of genotypes with different *Rpv* profiles revealed substantial differences in both sporulation and necrosis responses. Accordingly, these results confirmed the correlation between genotypic and phenotypic data, highlighting a specific symptomatology related to the presence of different resistant loci.

Sporulation and necrosis scores, assessed with two independent scales, did not show a general relation, even if a different correlation level between the two symptoms in the different *Rpv* classes was highlighted. For example, a positive and significant correlation was evident in the leaf discs of genotypes with *Rpv3-1*: poor or absent sporulation symptoms were observed along with scarce or no necrotic evidence. Instead, in discs of genotypes having both *Rpv3-3* and *Rpv10* loci, the correlation was negative: abundant necrotic flecks/spots (score one or three) were present along with poor or absent sporulation evidence (score seven or nine). As in our research, Blasi et al. [14] reported a positive correlation between sporulation and necrosis in their *Rpv* segregating population, while Divilov et al. [15] reported contrasting results. Some authors demonstrated that in the field many *P. viticola* strains exist that change over time [16] and that can determine dissimilar rates of sporulation and necrosis on the same genotype [13,17]. Our bioassays confirmed that in complex biologic situations, which comprise many hosts and/or pathogen strains, it is important to classify separately sporulation and necrosis reactions separately to well characterize the pathosystems and their related aspects.

In detail, genotypes of *Rpv-* class (Raboso Piave, RPxK_153 and RPxS_006, Glera and Chardonnay) showed similar symptoms in the experiments: a high level of sporulation (score 1 to 5) occurred with poor or absent necrosis (score 5 to 9), as reported for susceptible plants [1,2].

Kozma 20-3 showed no sporulation and only a few necrotic spots in a couple of leaf discs. Despite this variety having been used in many breeding programs providing several progenies, few data are available on its symptomatology in response to *P. viticola*. However, its carries resistance sources are quite well described in other varieties like Bianca and Regent for *Rpv3-1* [5,8,18] and Kunbarat and other accessions carrying *Rpv12* [6]. *Rpv3-1* is responsible for the onset of a hypersensitive response (HR) at the infection sites, it did not halt pathogen growth, but is associated with a significant reduction of pathogen performance and disease symptoms from 3 to 6 dpi [5]. *Rpv12* conferred the ability to establish an HR in type and timing like those triggered by *Rpv3-1*, but the limitation imposed on pathogen sporulation was more significant [6]. In our case, progenies carrying *Rpv3-1* and *Rpv12* exhibited different rating of resistance: when the *Rpv* alleles of both loci were combined there were no or few symptoms, as observed for Kozma 20-3; when only one resistance source was present (*Rpv3-1* or *Rpv12*) the pathogen control was slightly lower, but still high. Our observations revealed no significant differences among genotypes having *Rpv3-1* or *Rpv12* profiles. An additive effect was found for the loci: genotypes with the *Rpv3-1*+*Rpv12* profile showed a very high and stable resistance response in both the experiments. As already reported by Foria et al. [19], responses to DM differed depending on the genetic background of *Rpv3-1* plants.

In this study, Solaris exhibited a very high capacity to reduce pathogen growth (sporulation score 9) in both experiments, while also showing intermediate values of necrotic symptoms (scores 5-7). As already reported, Solaris confers downy mildew resistance accompanied by necrosis [20], callose deposition [21] and stilbene accumulation [22]. In the presence of high infection pressure, its resistance was demonstrated to be incomplete with tissue discolorations larger than those produced on Regent [23]. Our data, as recently reported by Vezzulli et al. [9], revealed the presence of two characterized resistance sources in Solaris (*Rpv3-3* and *Rpv10*) instead of one (*Rpv10*) as previously described [10]. In our work, the analyzed progenies of Solaris exhibiting different intensities of resistance expression in terms of both sporulation and necrosis. Nevertheless, all F1s showed a less effective defense response in containing DM growth than the one observed in the resistant parent. This is the case, as expected, of the genotypes carrying only *Rpv3-3* or *Rpv10*, but also the *Rpv3-3*+*Rpv10* progenies surprisingly exhibited only a medium resistance level with severe necrosis. Our data showed unsatisfactory expression of resistance for genotypes carrying only *Rpv10* resistant alleles, in contrast with other phenotypic evaluations conducted on *Rpv10* varieties such as Muscaris and Cabernet Cortis [24]. *Rpv3-3* genotypes showed evident necrotic symptoms in response to *P. viticola* infection, together with intermediate sporulation. In addition, *Rpv3-3* genotypes exhibited weaker resistance levels than *Rpv3-1* ones, as already evidenced by Foria et al. [19]. For partially resistant genotypes, the influence of the pathogen strain and natural host–pathogen interaction could be determinant in the resistance outcome. However, we speculated that other genetic determinants influencing the DM resistance in the progenies could be present in Solaris and our in-field evaluations (data not shown) supported the hypothesis.

The results of the LMMs clearly confirmed that “*Rpv* class” factor influenced plant necrosis and pathogen sporulation. Despite the “experiment” factor was significant only on pathogen growth, the low standard deviation for the “genotype” factor in sporulation LMM and the high residual deviation in necrosis LMM suggested that other not controlled elements affected the plants necrotic response.

In our study, the MAS was useful to select the most resistant genotypes early (*Rpv3-1*; *Rpv12; Rpv3-1*+*Rpv12*); in other cases, several *Rpv*+ seedlings that showed scarce aptitude in limiting the pathogen diffusion in artificial inoculation (*Rpv3-3*; *Rpv10*; *Rpv3-3*+*Rpv10*) were retained. In the case of *Rpv3-3*+*Rpv10* progenies of Solaris, additional considerations should be made: the control of *P. viticola* proliferation is consistent, but the leaf injury caused by severe necrotic responses is very important. How and to what extent this phenomenon affects leaf physiology should be clarified. On contrary the presence of two resistance sources *Rpv3-1* and *Rpv12* confirmed that the pyramiding ensure a higher degree of resistance and a more stable and durable trait [11].

Finally, it is evident that evaluating sporulation and necrosis symptoms separately is crucial to study in depth different resistance sources and mechanisms, also in genotypes carrying the same resistance profile. However, these observations should be confirmed broadening the number of individuals as well as pathogen isolates taking the experimental conditions carefully into account.

## 4. Materials and Methods

### 4.1. Segregating Populations and Varieties under Study

Raboso Piave, a minor *V. vinifera* variety susceptible to downy mildew, was crossed in 2016 at CREA - Research Centre for Viticulture and Enology, Susegana, Treviso, Italy (N 45.852098, E 12.255842). The resistant pollen donor hybrids were Kozma 20-3, carrying both *Rpv3-1* and *Rpv12* alleles associated with *P. viticola* resistance and Solaris, having both *Rpv3-3* and *Rpv10* resistance haplotypes. The two cross populations “RPxK” from Kozma 20-3 and “RPxS” from Solaris were MAS-selected and planted in field in 2017. Original F1s plantlets, the parental varieties and some susceptible *V. vinifera* were cultivated in the same plot without any fungicide treatment.

A representative set of 26 seedlings (13 from each population) carrying different *Rpv* loci, as well as the parental plants Raboso Piave, Kozma 20-3 and Solaris and the varieties Chardonnay and Glera were further studied by some phenotyping bioassay. The list of analyzed genotypes according to their *Rpv* profile is shown in Table S3.

### 4.2. Genotyping MAS

RPxK and RPxS populations, including the parents as well as the control *V. vinifera* varieties, were analyzed for the presence of resistance haplotypes by using SSR markers as described in De Nardi et al. [25], according to their expected resistance sources (*Rpv3-1*, *Rpv3-3*, *Rpv10* and *Rpv12* loci). Genomic DNA was extracted from about 50 mg of young leaf tissue using “DNeasy 96 Plant Kit” (Qiagen, Hilden, Germany). PCR reactions were performed in a 10 μL volume containing 200 μM of each dNTP, 0.024 to 0.176 μM of each primer and 0.25 U of Taq DNA polymerase (MyTaq, Bioline, UK). PCR were performed under the following thermal profile: 94 °C for 4 min, followed by 35 cycles at 94 °C for 45 s, 56 °C for 1 min 30 s, 72 °C for 1 min and final elongation of 20 min at 72 °C. The PCR products were separated by capillary electrophoresis using an ABI Prism 3130xl DNA analyzer (Life Technologies, Foster City, CA, USA). The SSR and alleles associated with resistance to *P. viticola* are reported in Table S1.

### 4.3. Phenotyping: Leaf Discs Infections

Plants phenotyping was performed by artificial inoculation of leaf discs in two independent experiments. Experiment A was conducted in the first ten days of July 2018 and experiment B in the last ten days of August 2018.

Briefly, from each in-field untreated plant, two fully expanded leaves with no evidence of foliar diseases were detached from the fourth-sixth nodes beneath the shoot apex. Six discs (18 mm diameter) per genotype were excised with a cork borer and transferred with the abaxial side up onto wet filter study in Petri dishes.

Fresh inoculum of *P. viticola* was prepared just before the bioassays from naturally infected leaves of *V. vinifera* varieties in the experimental vineyard at two different times. For each experiment, fresh sporulated leaves were soaked in sterile water to obtain an inoculum of 1 x 10^6^ sporangia/mL. The suspension was sprayed on the abaxial leaf surface of four discs per genotype. Mock inoculation (distilled water) was performed on the remaining two discs. Sealed Petri dishes were incubated at 23 °C with high relative humidity for 9 days. In the first 24 h they were maintained in the dark, then under 16/8 h (light/dark) photoperiod.

Disease symptoms on leaf discs were visually evaluated at 9 dpi (days post-inoculation). Sporulation and necrosis/browning were considered separately, by applying different scores for pathogen reaction and host reaction to each disc (Figure 1). Both scoring parameters used five classes, detailed as follows. For sporulation: 1 = strong sporulation in unlimited patches; 3 = abundant sporulation in vast patches; 5 = moderate sporulation with medium-size patches; 7 = low sporulation in limited patches; 9 = no sporulation. For necrosis/browning: 1 = continuous and/or big flecks, 3 = numerous flecks, 5 = numerous spots and/or few necrotic flecks, 7 = few necrotic spots, 9 = no necrosis/browning.

### 4.4. Statistical Analysis

For each experiment (A and B), sporulation and necrosis scoring were assigned to each genotype as the mean of the four inoculated discs. Only the 26 progenies and the varieties Chardonnay and Glera values were considered in the statistical analysis. The data for sporulation and necrosis were used separately to fit two linear mixed effect models (LMM), where “*Rpv* class” and “Experiment” variables were considered as fixed factors while “Genotype” as random factor. The optimal model structure was chosen according to the strategy described in Zurr et al. [26]. Pairwise comparisons were performed following Tukey’s method.

Correlation between sporulation and necrosis reactions at leaf disc level was evaluated with the Kendall rank correlation coefficient (TAU) using the scores from inoculated leaf discs.

Statistical analyses were performed using R *v*3.5.2 [27] with the *lme4 v*1.1-20 package [28] to fit the LMMs and the *emmeans v*1.3.2 package [29] for pairwise comparisons.

## 5. Conclusions

In conclusion, for breeding purposes, we suggest taking care in selecting the type, origin and effectiveness of resistance sources to introgress, also in combination. Indeed, as reported also in this research, the resistance level exhibited by the ancestral/parental line could be the results of known, but also unknown genetic factors, which could be lost in the progenies. For these reasons, accurate and very early phenotyping of segregating populations is fundamental, especially in the presence of not well-characterized resistance determinants. In these cases, MAS analysis alone could not be resolutive in selecting the most resistant genotypes.

The phenotyping needs to be standardized and repeated to well-characterize the pathosystem and explain the variability shown by the genotypes. Moreover, different factors that affect experiment development and results must be considered also based on phenotyping purposes: different inoculates, plant material and environmental conditions.

## Supplementary Materials

The following are available online at https://www.mdpi.com/2223-7747/9/6/781/s1, **Figure S1.** Leaf discs records for sporulation and necrosis at 9 dpi from the two phenotyping experiments for the different studied genotypes. Chardonnay, Glera and Raboso Piave are three *V. vinifera* varieties sensitive to *P. viticola* while Kozma 20/3 and Solaris two resistant ones. “RPxK_n” and “RpxS_n” are progenies of the crosses Raboso Piave x Kozma 20/3 and Raboso Piave x Solaris respectively, segregating for the resistance loci carried by the parental plants. Point’s sizes are related to the number of discs showing the same combinations of symptoms as reported in the legend. Colors grouped the genotypes by resistance class. **Table S1.** The parental plants of the progenies together with their carried *Rpv* loci. Subsequently, the markers associated to the loci utilized for the marker assisted selection (MAS) and in bold and underlined the resistance associate allele haplotypes. **Table S2.** Segregation numbers and MAS selected progenies. **Table S3.** Genotypes characterized in the study. “RPxK_n” and “RpxS_n” are progenies of the crosses Raboso Piave x Kozma 20/3 and Raboso Piave x Solaris respectively. The mean sporulation and necrosis values for two independent experiments was calculated at 9 dpi from four leaf discs each. **Table S4.** Statistics of the fitted linear mixed models (LMM). As intercept were defined the class *Rpv*- and the Exp. A. The “Estimate mean” describes the effects (positives or negatives) of the fixed factors on sporulation and necrosis scores taking as reference the intercept.

## References

[1] Díez-Navajas A.M., Wiedemann-Merdinoglu S., Greif C., Merdinoglu D. (2008). Nonhost Versus Host Resistance to the Grapevine Downy Mildew, *Plasmopara viticola*, Studied at the Tissue Level. Phytopathology.

[2] Cadle-Davidson L. (2008). Variation within and between *Vitis* spp. for Foliar Resistance to the Downy Mildew Pathogen *Plasmopara viticola*. Plant Dis..

[3] Maul E., Sudharma K.N., Ganesh A., Hundemer M., Walk M., vom Weg S., Mahler-Ries A., Brühl U., Töpfer R. Vitis International Variety Catalogue. www.vivc.de.

[4] Di Gaspero G., Copetti D., Coleman C., Castellarin S.D., Eibach R., Kozma P., Lacombe T., Gambetta G., Zvyagin A., Cindrić P. (2012). Selective Sweep at the *Rpv3* Locus during Grapevine Breeding for Downy Mildew Resistance. Theor. Appl. Genet..

[5] Bellin D., Peressotti E., Merdinoglu D., Wiedemann-Merdinoglu S., Adam-Blondon A.F., Cipriani G., Morgante M., Testolin R., Di Gaspero G. (2009). Resistance to *Plasmopara viticola* in Grapevine ‘Bianca’ Is Controlled by a Major Dominant Gene Causing Localised Necrosis at the Infection Site. Theor. Appl. Genet..

[6] Venuti S., Copetti D., Foria S., Falginella L., Hoffmann S., Bellin D., Cindrić P., Kozma P., Scalabrin S., Morgante M. (2013). Historical Introgression of the Downy Mildew Resistance Gene *Rpv12* from the Asian Species *Vitis amurensis* into Grapevine Varieties. PLoS ONE.

[7] Fischer B.M., Salakhutdinov I., Akkurt M., Eibach R., Edwards K.J., Töpfer R., Zyprian E.M. (2004). Quantitative Trait Locus Analysis of Fungal Disease Resistance Factors on a Molecular Map of Grapevine. Theor. Appl. Genet..

[8] van Heerden C.J., Burger P., Vermeulen A., Prins R. (2014). Detection of Downy and Powdery Mildew Resistance QTL in a ‘Regent’ × ‘RedGlobe’ Population. Euphytica.

[9] Vezzulli S., Malacarne G., Masuero D., Vecchione A., Dolzani C., Goremykin V., Mehari Z.H., Banchi E., Velasco R., Stefanini M. (2019). The *Rpv3-3* Haplotype and Stilbenoid Induction Mediate Downy Mildew Resistance in a Grapevine Interspecific Population. Front. Plant Sci..

[10] Schwander F., Eibach R., Fechter I., Hausmann L., Zyprian E., Töpfer R. (2012). *Rpv10*: A New Locus from the Asian *Vitis* Gene Pool for Pyramiding Downy Mildew Resistance Loci in Grapevine. Theor. Appl. Genet..

[11] Eibach R., Zyprian E., Welter L., Töpfer R. (2007). The Use of Molecular Markers for Pyramiding Resistance Genes in Grapevine Breeding. Vitis.

[12] OIV (2009). Descriptor List for Grape Varieties and Vitis Species.

[13] Gómez-Zeledón J., Kaiser M., Spring O. (2016). An Extended Leaf Disc Test for Virulence Assessment in *Plasmopara viticola* and Detection of Downy Mildew Resistance in *Vitis*. J. Plant Pathol. Microbiol..

[14] Blasi P., Blanc S., Wiedemann-Merdinoglu S., Prado E., Rühl E.H., Mestre P., Merdinoglu D. (2011). Construction of a Reference Linkage Map of *Vitis amurensis* and Genetic Mapping of *Rpv8*, a Locus Conferring Resistance to Grapevine Downy Mildew. Theor. Appl. Genet..

[15] Divilov K., Barba P., Cadle-Davidson L., Reisch B.I. (2018). Single and Multiple Phenotype QTL Analyses of Downy Mildew Resistance in Interspecific Grapevines. Theor. Appl. Genet..

[16] Matasci C.L., Jermini M., Gobbin D., Gessler C. (2010). Microsatellite Based Population Structure of *Plasmopara viticola* at Single Vine Scale. Eur. J. Plant Pathol..

[17] Delmotte F., Mestre P., Schneider C., Kassemeyer H.H., Kozma P., Richart-Cervera S., Rouxel M., Delière L. (2014). Rapid and Multiregional Adaptation to Host Partial Resistance in a Plant Pathogenic Oomycete: Evidence from European Populations of *Plasmopara viticola*, the Causal Agent of Grapevine Downy Mildew. Infect. Genet. Evol..

[18] Welter L.J., Göktürk-Baydar N., Akkurt M., Maul E., Eibach R., Töpfer R., Zyprian E.M. (2007). Genetic Mapping and Localization of Quantitative Trait Loci Affecting Fungal Disease Resistance and Leaf Morphology in Grapevine (*Vitis vinifera* L). Mol. Breed..

[19] Foria S., Magris G., Morgante M., Di Gaspero G. (2018). The Genetic Background Modulates the Intensity of *Rpv3*-Dependent Downy Mildew Resistance in Grapevine. Plant Breed..

[20] Boso S., Kassemeyer H.H. (2008). Different Susceptibility of European Grapevine Cultivars for Downy Mildew. Vitis.

[21] Gindro K., Pezet R., Viret O. (2003). Histological Study of the Responses of Two *Vitis vinifera* Cultivars (Resistant and Susceptible) to *Plasmopara viticola* Infections. Plant Physiol. Biochem..

[22] Pezet R., Gindro K., Viret O., Spring J.L. (2004). Glycosylation and Oxidative Dimerization of Resveratrol Are Respectively Associated to Sensitivity and Resistance of Grapevine Cultivars to Downy Mildew. Physiol. Mol. Plant Pathol..

[23] Oerke E.C., Herzog K., Toepfer R. (2016). Hyperspectral Phenotyping of the Reaction of Grapevine Genotypes to *Plasmopara viticola*. J. Exp. Bot..

[24] Vezzulli S., Vecchione A., Stefanini M., Zulini L. (2018). Downy Mildew Resistance Evaluation in 28 Grapevine Hybrids Promising for Breeding Programs in Trentino Region (Italy). Eur. J. Plant Pathol..

[25] De Nardi B., Santellani F., Possamai T., Velasco R. (2019). Breeding for Mildew Resistance in Grapevine to Improve Environmental and Socio-Economic Sustainability in Hotspot Areas of Veneto. Acta Hortic..

[26] Zuur A.F., Ieno E.N., Walker N.J., Saveliev A.A., Smith G.M., Walker Z.I., Smith S. (2009). Mixed Effects Modelling for Nested Data. Mixed Effects Models and Extensions in Ecology with R.

[27] R Core Team (2019). R: A Language and Environment for Statistical Computing. R Foundation for Statistical Computing, Vienna, Austria. https://www.R-project.org.

[28] Bates D., Maechler M., Bolker B., Walker S., Christensen R.H.B., Singmann H., Dai B., Eigen C. (2015). Lme4: Fitting Linear Mixed-Effects Models Using Lme4. J. Stat. Softw..

[29] Russell L. (2019). Emmeans: Estimated Marginal Means, Aka Least-Squares Means. R Package Version 1.3.2. https://cran.r-project.org/web/packagies/emmeans.

