# Supplementary material for: Rpv Mediated Defense Responses in Grapevine Offspring Resistant to Plasmopara viticola
Source: Plants (Basel). 2020 Jun 22;9(6):781. doi: 10.3390/plants9060781 (PMC7356695; doi:10.3390/plants9060781)
Supplement: Supplementary file 1 [file plants-09-00781-s001.pdf]

plants

MDPI

Supplementary Materials

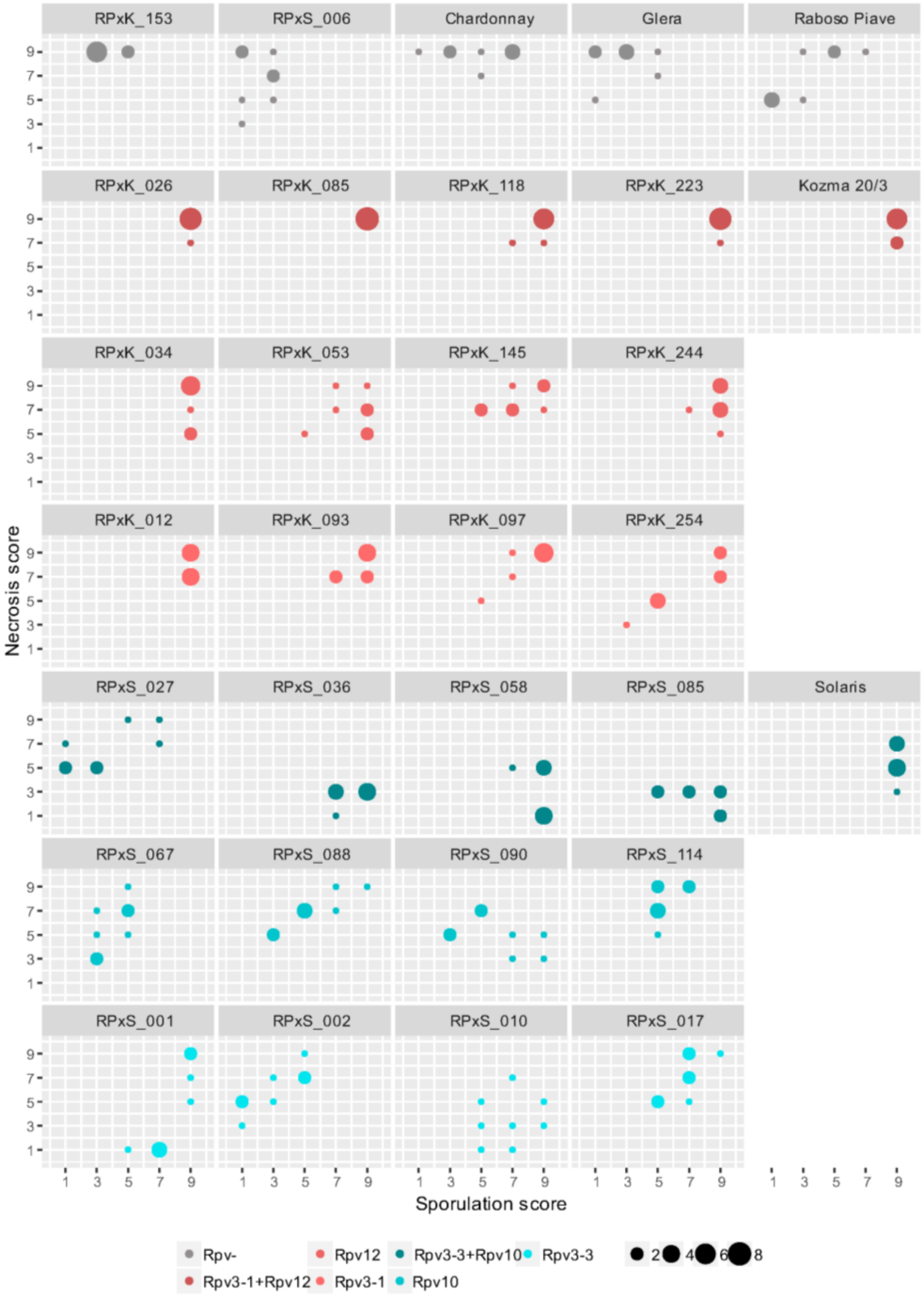

Figure S1. Leaf discs records for sporulation and necrosis at 9 dpi from the two phenotyping experiments for the different studied genotypes. Chardonnay, Glera and Raboso Piave are three *V. vinifera* varieties sensitive to *P. viticola* while Kozma 20/3 and Solaris two resistant ones. "RPxK_n" and "RpxS_n" are progenies of the crosses Raboso Piave x Kozma 20/3 and Raboso Piave x Solaris respectively, segregating for the resistance loci carried by the parental plants. Point's sizes are related to the number of discs showing the same combinations of symptoms as reported in the legend. Colors grouped the genotypes by resistance class.

Table S1. The parental plants of the progenies together with their carried *Rpv* loci. Subsequently, the markers associated to the loci utilized for the marker assisted selection (MAS) and in bold and underlined the resistance associate allele haplotypes.

Parental Plants	Resistance Loci	Markers Associated to the Loci	Parental Alleles	Reference for the Loci	Marker Sequences Forward and Reverse Primers	
Kozma 20-3	*Rpv3-1*	UDV305; UDV737; VMC7f2	**299**/356 **282**/297 **211**/200	[4,5]	TGGTGCAATGGTCATAATTT<br>TTTGCATGCGATACCTGAAGC<br>AAGAAAGTTTGCAGTTTATGGTG	GAGGAAAAGAGAAAGCAAAGA<br>TCCTGCAGCTGTTGACGATA<br>AAGATGACAATAGCGAGAGAGAA
	Rpv12	UDV350; UDV360	**308**/302 **208**/201	[6]	TTTTGGGAGTTTCCATGTCC<br>TGCTTTACAGGTGACCATCAA	AAGACACCTTGGGGGATAAAA<br>GCAACCAATTGAGGGGATTA
Solaris	*Rpv3-3*	UDV305; UDV737; VMC7f2	**null**/334 **274**/307 **200**/198	[4,5]	TGGTGCAATGGTCATAATTT<br>TTTGCATGCGATACCTGAAGC<br>AAGAAAGTTTGCAGTTTATGGTG	GAGGAAAAGAGAAAGCAAAGA<br>TCCTGCAGCTGTTGACGATA<br>AAGATGACAATAGCGAGAGAGAA
	Rpv10	Gf09-44; Gf09-46; Gf09-47	**232**/248 **410**/419 **297**/294	[10]	CATCGTTCCTTTCTTACTCGCT<br>GAGAGATTTGAGGGATTGTTGG<br>CCACATTCTTCCTGCACATAAA	GCTAATGGAGGGTAGTGCTCAA<br>ATCCACGTTTGTAGCCTTTTGT<br>CTGTTGTAAGGGCTCCCAATTA
Raboso Piave	*Rpv-*	//	//	//		

Table S2. Segregation numbers and MAS selected progenies.

Population	Seedlings	Two Loci %		One Locus %		No Loci %		Maintained Plants
RPxK	224	*Rpv3-1+ Rpv12*	13.27	*Rpv3-1*	*20,85*	*Rpv-*	45.97	110
				Rpv12	*19,91*			
RPxS	538	*Rpv3-3+ Rpv10*	23.20	*Rpv3-3*	*27,10*	*Rpv-*	28.07	255
				Rpv10	*21.64*			

Table S3. Genotypes characterized in the study. "RPxK_n" and "RpxS_n" are progenies of the crosses Raboso Piave x Kozma 20/3 and Raboso Piave x Solaris respectively. The mean sporulation and necrosis values for two independent experiments was calculated at 9 dpi from four leaf discs each.

Genotype ID	*Rpv* Class[a]	Exp.	Sporulation Mean Score	Sporulation Frequency	Necrosis Mean Score	Necrosis Frequency
Chardonnay	*Rpv-*	A	4.0	1.00	9.0	0.00
Chardonnay	*Rpv-*	B	5.5	1.00	8.5	0.25
Glera	*Rpv-*	A	1.5	1.00	8.0	0.25
Glera	*Rpv-*	B	4.0	1.00	8.5	0.25
Raboso Piave	*Rpv-*	A	1.5	1.00	5.0	1.00
Raboso Piave	*Rpv-*	B	5.0	1.00	9.0	0.00
Kozma 20/3	*Rpv3-1+Rpv12*	A	9.0	0.00	8.5	0.25
Kozma 20/3	*Rpv3-1+Rpv12*	B	9.0	0.00	8.5	0.25
Solaris	*Rpv3-3+Rpv10*	A	9.0	0.00	5.5	1.00
Solaris	*Rpv3-3+Rpv10*	B	9.0	0.00	5.5	1.00
RPxK_153	*Rpv-*	A	3.5	1.00	9.0	0.00
RPxK_153	*Rpv-*	B	3.5	1.00	9.0	0.00
RPxS_006	*Rpv-*	A	2.0	1.00	8.5	0.25
RPxS_006	*Rpv-*	B	2.0	1.00	5.0	1.00
RPxK_034	*Rpv12*	A	9.0	0.00	9.0	0.00
RPxK_034	*Rpv12*	B	9.0	0.00	6.5	0.75
RPxK_053	*Rpv12*	A	8.5	0.25	7.5	0.50
RPxK_053	*Rpv12*	B	8.0	0.50	6.0	1.00
RPxK_145	*Rpv12*	A	7.0	0.75	8.0	0.50
RPxK_145	*Rpv12*	B	8.0	0.50	7.5	0.75
RPxK_244	*Rpv12*	A	9.0	0.00	8.5	0.25
RPxK_244	*Rpv12*	B	8.5	0.25	6.5	1.00
RPxK_012	*Rpv3-1*	A	9.0	0.00	7.5	0.75
RPxK_012	*Rpv3-1*	B	9.0	0.00	8.5	0.25
RPxK_093	*Rpv3-1*	A	8.0	0.50	7.0	1.00
RPxK_093	*Rpv3-1*	B	9.0	0.00	9.0	0.00
RPxK_097	*Rpv3-1*	A	7.0	0.75	7.5	0.50
RPxK_097	*Rpv3-1*	B	9.0	0.00	9.0	0.00
RPxK_254	*Rpv3-1*	A	4.5	1.00	4.5	1.00
RPxK_254	*Rpv3-1*	B	9.0	0.00	8.0	0.50
RPxK_026	*Rpv3-1+Rpv12*	A	9.0	0.00	9.0	0.00
RPxK_026	*Rpv3-1+Rpv12*	B	9.0	0.00	8.5	0.25
RPxK_085	*Rpv3-1+Rpv12*	A	9.0	0.00	9.0	0.00
RPxK_085	*Rpv3-1+Rpv12*	B	9.0	0.00	9.0	0.00
RPxK_118	*Rpv3-1+Rpv12*	A	8.5	0.25	8.0	0.50
RPxK_118	*Rpv3-1+Rpv12*	B	9.0	0.00	9.0	0.00
RPxK_223	*Rpv3-1+Rpv12*	A	9.0	0.00	8.5	0.25
RPxK_223	*Rpv3-1+Rpv12*	B	9.0	0.00	9.0	0.00
RPxS_067	*Rpv10*	A	3.5	1.00	4.0	1.00
RPxS_067	*Rpv10*	B	4.5	1.00	7.5	0.75
RPxS_088	*Rpv10*	A	5.5	0.75	7.0	0.75
RPxS_088	*Rpv10*	B	5.5	1.00	7.0	0.75
RPxS_090	*Rpv10*	A	4.0	1.00	6.0	1.00
RPxS_090	*Rpv10*	B	8.0	0.50	4.0	1.00
RPxS_114	*Rpv10*	A	6.0	1.00	9.0	0.00
RPxS_114	*Rpv10*	B	5.5	1.00	7.0	1.00
RPxS_001	*Rpv3-3*	A	6.5	1.00	1.0	1.00
RPxS_001	*Rpv3-3*	B	9.0	0.00	7.5	0.50
RPxS_002	*Rpv3-3*	A	3.0	1.00	6.0	1.00
RPxS_002	*Rpv3-3*	B	3.0	1.00	7.0	0.50
RPxS_010	*Rpv3-3*	A	5.5	1.00	4.0	1.00
RPxS_010	*Rpv3-3*	B	8.0	0.50	3.0	1.00
RPxS_017	*Rpv3-3*	A	6.5	0.75	6.5	0.75

RPxS_017	*Rpv3-3*	B	7.0	1.00	7.5	0.50
RPxS_027	*Rpv3-3+Rpv10*	A	5.0	1.00	6.5	0.75
RPxS_027	*Rpv3-3+Rpv10*	B	3.0	1.00	6.5	0.75
RPxS_036	*Rpv3-3+Rpv10*	A	7.0	1.00	2.5	1.00
RPxS_036	*Rpv3-3+Rpv10*	B	9.0	0.00	3.0	1.00
RPxS_058	*Rpv3-3+Rpv10*	A	9.0	0.00	1.0	1.00
RPxS_058	*Rpv3-3+Rpv10*	B	8.5	0.25	5.0	1.00
RPxS_085	*Rpv3-3+Rpv10*	A	6.0	1.00	3.0	1.00
RPxS_085	*Rpv3-3+Rpv10*	B	9.0	0.00	2.0	1.00

[a] according to the presence of SSR alleles associated to the analyzed *Rpv*-resistances.

Table S4. Statistics of the fitted linear mixed models (LMM). As intercept were defined the class *Rpv-* and the Exp. A. The "Estimate mean" describes the effects (positives or negatives) of the fixed factors on sporulation and necrosis scores taking as reference the intercept.

	**LMM Sporulation**		**LMM Necrosis**	
**Predictors**	**Estimate Mean**	**Std. Error**	**Estimate Mean**	**Std. Error**
Intercept - (*Rpv-*; Exp. A)	2.81	0.68	8.02	0.64
Rpv10	2.06	0.94	-1.75	0.86
Rpv3-3	2.81	0.94	-2.88	0.86
Rpv3-3+Rpv10	3.81	0.94	-4.50	0.86
Rpv3-1	4.81	0.94	-0.56	0.86
Rpv12	5.13	0.94	-0.75	0.86
Rpv3-1+Rpv12	5.69	0.94	0.56	0.86
Exp. B	0.88	0.28	0.34	0.41
**Random Effect**				
Genotype std. dev.	1.10		0.56	
Residual std. dev.	1.07		1.53	
**p from F Test on Fixed Effects (Satterthwaite Approximation)**				
Resistance class	0.0001		0.0001	
Experiment	0.0048		0.4133	
